# Characterizing individuals accessing mental health services in the UAE: a focus on youth living in Dubai

**DOI:** 10.1186/s13033-021-00452-4

**Published:** 2021-03-31

**Authors:** Mariapaola Barbato, Shaikha Al Hemeiri, Shorouk Nafie, Baraa A. Dhuhair, Nadia T. Dabbagh

**Affiliations:** 1 Department of Psychology, College of Natural and Health Sciences, Zayed University, Dubai, UAE; 2 Al Jalila Children’s Specialty Hospital, Dubai, UAE; 3 Student Counseling Center, Zayed University, Dubai, UAE; 4 Child Psychiatry Service, Psychiatry Department, Rashid Hospital, Dubai Health Authority, Dubai, UAE

**Keywords:** Mental health, Youth, UAE, Prevention, Multi‐cultural, Immigration, Expat

## Abstract

**Background:**

Most mental health issues develop during adolescence, therefore identifying youth mental health needs and pathways to care is critical to improve prevention. To date, studies have typically focused on Western samples, while the impact of cultural diversity on perception of health and illness, and pathways to care, remain poorly understood. To address the shortage of studies conducted in the Arab world, and particularly in the United Arab Emirates (UAE), the present investigation aims to identify the characteristics of youth accessing mental health services in Dubai.

**Methods:**

Data was collected retrospectively from patients’ records at Rashid Hospital Child Psychiatry Service. Information collected included demographics, life stressors, symptoms duration, main diagnosis, and presence/absence of psychotic features in patients’ symptomatology. The relationship between demographic and clinical variables was explored using *Chi-square* tests and negative binomial regression models.

**Results:**

The sample included 99 treatment-seeking young patients (mean age 15.3; SD = 1.7); 47.5 % were Emirati (UAE national) and 52.5 % were non-Emirati patients. In our treatment-seeking youth sample Depressive disorders represented the most frequent diagnosis, followed by Bipolar and related disorders, Anxiety and stress related disorders, and Schizophrenia and psychotic disorders. Compared to Emirati patients, non-Emirati patients were more likely to report relationships with friends as a source of stress. Female help-seekers, compared to males, were more likely to report stressful relationships with family members, and to receive a diagnosis of Depressive disorders. The duration of symptoms before seeking help was significantly predicted by family stress, gender, self-harm behavior, a symptomatology with psychotic features, and a diagnosis of Anxiety disorders.

**Conclusions:**

The present study contributes to characterizing youth accessing mental health services at Rashid Hospital’s Child Psychiatry service in Dubai. An overall prevalence of poor family functioning among help-seeking youth, and the importance of peer support for expatriate youth were highlighted. Gender differences in perceived stressors, diagnoses and help-seeking behavior suggest the need to promote help-seeking among young boys. While presentation with psychotic features seems to lead to quicker access to medical care, self-harm and anxiety appear to delay help-seeking. The potential implications of our results for promoting youth wellbeing in the region are discussed.

## Background

Most mental health issues develop during adolescence and early adulthood, impacting youth in several life domains [1]. There is a growing consensus that early detection, prevention and intervention during adolescence can help reduce the burden of mental illness [2–4]. To this end, understanding the characteristics, perceived stressors, and pathways to care of youth who develop mental illness is critical to early detection and treatment. However, there are several challenges that need to be overcome. For instance, access to child and adolescent mental health services is typically low [5], posing an obstacle to understanding the needs of this population. Moreover, while the current understanding of mental illness has emerged from purely Western perspectives, there is now general agreement on the impact of cultural diversity on perception of health and illness, help-seeking behavior and pathways to care [6]. With the current rise of globalization and large waves of immigration from low to high-income countries, understanding the differential impact of culture-specific factors such as stigma, spirituality, or shame in the development of mental illness seems imperative [7, 8].

The United Arab Emirates (UAE), a federation of seven states on the east coast of the Arabian Peninsula, has a rapidly growing population, one-third of which is below 25 years of age [9]. In the past few decades the country has gone through rapid urbanization, leading to significant changes in societal structure and a dramatic rise in mental health concerns [10]. Importantly, the UAE has a population of 9.6 million individuals, 8 million of whom are foreigners from all around the world [11]. Recently, it has become increasingly evident that the stress of migration represents a potential threat to the mental health of migrants and their children [12–16]. Several studies have highlighted the challenges of child and adolescent adjustment to a foreign environment (e.g. [17, 18]), as well as the impact of acculturative stress on adolescent mental health [19, 20]. For these reasons, identifying the mental health needs and pathways to care of youth living in the UAE seems crucial to improve their life trajectories.

Studies conducted in the UAE are sparse. Some studies have focused on UAE national samples only, and many only included non-help-seeking samples. The few studies conducted on youth have highlighted a high prevalence of mental illness [21] with depression [22, 23], anxiety [21] and eating disorders [24–26] being prevalent. A reluctance to seek help due to stigma and scepticism regarding the efficacy of conventional Western medical treatment has also been reported [27–29]. A few studies have focused specifically on UAE migrant/expatriate samples, reporting higher rates of suicide among adult expatriates compared to UAE nationals [30], and an increased frequency of risky behaviors among adolescents [31].

Overall, although sparse, the literature looking at youth mental health in the UAE has highlighted a high prevalence of mental health problems, confirming the need to enhance prevention and early intervention in the region. To this end, a better understanding of the role played by various factors such as gender or immigration status on symptom development and help-seeking choices is essential.

The present study aimed to describe a sample of young individuals accessing mental health services in the UAE, with the goal of better understanding pathways to professional medical care. More specifically, the first aim of the study was to describe our sample by looking at demographic characteristics (gender and residential status), types of stressors experienced, frequency of self-harm and suicidal behavior, and clinical diagnosis. A second aim of the study was to explore the relationship between residential status, frequency of stressors and clinical diagnoses, as well as between gender, frequency of stressors and clinical diagnoses. A third aim of the present study was to identify potential predictors of symptoms duration (i.e., number of days) before seeking help. To the best of our knowledge, our study represents the first attempt to describe youth accessing mental health services in Dubai.

## Method

The current study was conducted at Rashid Hospital’s Child Psychiatry service, Psychiatry Department, Dubai Health Authority, Dubai. The Trauma Centre at Rashid Hospital has the main Emergency Department that serves the Emirate of Dubai and the northern Emirates.

The study was approved by the relevant Institutional Review Board (Dubai Health Authority: DSREC-02/2019_13; Zayed University: ZU18_120_E). Psychiatry Case Records for patients referred to Child Psychiatry between 2011 and 2016 were accessed retrospectively. Case Records included information collected from patients and/or their family members at the time of intake. Information was collected in English or Arabic, based on the patient’s or family member’s preference. Occasionally, some assessments had been conducted in other languages (e.g., Hindi) upon patients’ requests and based on staff availability. Patients were included if their age at the time of first intake was between 12 and 25, if they lived in the UAE, and if they received a diagnosis of Schizophrenia Spectrum and Other Psychotic Disorders, Bipolar and Related Disorders, Depressive Disorders, Anxiety Disorders, Obsessive Compulsive Disorder or Trauma and Stress Related Disorders, based on ICD-10 diagnostic criteria. Patients were excluded if they had a co-existing neurological disorder or if symptoms could be explained by another serious medical condition.

Data extracted from case records included demographics (age, gender, nationality as per patient’s ID), presence/absence of deliberate self-harm and suicidal thoughts, family psychiatric history (Yes/No), main diagnosis, presence/absence of psychotic features in the patient’s symptomatology. Perceived life stressors possibly triggering the symptoms, as reported in the case forms, were grouped in five categories: Family (e.g., parents’ divorce), Friends (e.g., best friend leaving the country), Academic Performance (e.g., failing tests or struggling with grades), Health (e.g., gastric problems) and Religion (e.g., lack of faith; for more details, see Table 1). Each category was considered as a dichotomous variable (Yes = 1; No = 0). Past psychiatric history included symptoms duration in days (i.e., the time between first onset of symptoms and first hospital intake).

**Table 1:** Stressors categories and examples

Stressor type	Examples
Family	Parents’ divorce, separation from one parent, poor relationship with a family member, conflict with stepmother/stepfather, abusive parent, alcohol dependence in the family, death of a family member, mental illness in the family
Friends	No friends, social isolation, death of a friend, fighting with best friend, friend moved abroad, bullying, difficulties with romantic relationships.
Academic	School change, decline in academic performance, failing in school, failing an exam to get into desired school, school drop out
Health	Hair loss, motor problems, kidney problems, gastric ulcer, recent surgery, puberty, frequent headaches, poor sleep, lack of appetite
Religion	Worry about death and judgment day, lack of faith, feelings of guilt.

### Data analysis

Statistical analyses were performed using SPSS version 26 (IBM, Armonk, NY, USA). Frequencies analysis was used to describe the demographic and clinical characteristics of our sample, as well as the frequency of stressors experienced. To compare symptoms duration across gender Mann-Whitney *U* test was used. To assess the impact of demographic differences on stressors experienced and clinical diagnosis, *Chi-square* test was used. Regression models for count data were used to identify predictors of symptoms duration (i.e., number of days) before help-seeking. Following Payne et al. [32] violation of the equidispersion assumption was assessed; the Chi-square/df ratio indicated overdispersion, hence a negative binomial regression model was selected over Poisson regression [32]. Potential predictors of symptoms duration entered in the model included gender, residential status (Emirati/non-Emirati), life stressors, self-harm behavior, suicidality, psychotic features, and main diagnosis.

## Results

The initial sample included 122 patients. Of these, 23 were later excluded due to either not meeting inclusion criteria or due to excessive missing data in the case record form. The final sample included 99 patients (age 12–19); 47.5 % were Emirati while the remainder were from different geographical regions including the Middle East (22 %), Asia (13 %), Africa (10 %), Europe (3 %), North America (3 %) and Australia (1 %). The demographic characteristics of the sample are shown in Table 2.

**Table 2:** Demographic characteristics

Variable	N = 99	
	Mean (SD)	
Age (years)	15.3 (1.7)	
	Frequency (%)	95 % C.I.
Sex Male Female	42 (42.4 %) 57 (57.6 %)	32.3–52.5 47.5–67.7
Nationality Emirati Middle Eastern Asian African European North American Australian	47 (47.5 %) 22 (22.2 %) 13 (13.1 %) 10 (10.1 %) 3 (3 %) 3 (3 %) 1 (1 %)	42.5–52.5 28.6–56.1 6.4–19.8 4–16.2 0–7.1 0–7.1 0–3.0
Diagnosis Anxiety disorders, obsessive compulsive disorder, trauma and stress related disorders Bipolar and related disorders Depressive disorders Schizophrenia spectrum and other psychotic disorders	21 (21.2 %) 23 (23.2 %) 42 (42.4 %) 13 (13.1 %)	13.1–29.3 16.2–31.3 32.3–52.5 7.1–20.2

The average symptoms duration in the overall sample was 409.97 days (SD = 870.36); symptoms duration did not differ significantly across gender (Males: M = 358.65; SD = 801.41; Females: M = 450.22; SD = 926.74; *U* = 894.00; p = 0.31). Looking at categories of stressors in the overall sample, Family was the most frequently reported (56.8 %) followed by Friends (38.4 %), Academic Performance (37.4 %), Health (9.1 %) and Religion (3 %). The frequency of stressors experienced by gender and residential status (Emirati/non-Emirati) is reported in Table 3. Non-Emirati patients were more likely than Emirati patients to report their relationships with friends as a source of stress (48.1 % vs. 27.7 %; *χ*^2^ = 4.35; *p* < 0.05). No group difference was observed between Emirati and non-Emirati patients in the frequency of Academic Performance stress (34 % vs. 40.4 %). The frequency of Family stress was higher among Emirati than non-Emirati patients (66 % vs. 51.9 %), although this difference did not reach statistical significance, possibly due to the small sample size. Across gender, in our sample Family stressors were more frequently experienced by females than males (Males: 45.2 %; Females: 68.4 %; *χ*^2^ = 5.36; p < 0.05); there was no significant gender difference in the frequency of stressors related to Academic Performance (Males: 35.7 %; Females: 38.6 %) or Friends (Males: 40.5 %; Females: 36.8 %). The relationship between gender, residential status, and stressors related to health and religion was not computed due to these stressors being reported only by a small minority of patients.

**Table 3:** Frequency of stressors by gender and residential status

Stressors	Gender % (95 % C.I.)		Residential status % (95 % C.I.)	
	Male N = 42	Female N = 57	Emirati N = 47	Non-Emirati N = 52
Family	45.2 (28.6, 61.8)	68.4 (56.1, 80.7)	66.0 (53.2, 68.7)	51.9 (38.5, 65.4)
Friends	40.5 (28.6, 54.8)	36.8 (24.6, 50.9)	27.7 (14.9, 40.4)	48.1 (34.6, 61.5)
Academic	35.7 (21.5, 50.0)	38.6 (26.3, 50.9)	34.0 (21.3, 46.8)	40.4 (28.8, 53.8)
Health	16.7 (7.1, 28.6)	3.5 (0.0, 8.8)	8.5 (2.1, 17.0)	9.6 (1.9, 17.3)
Religion	2.4 (0.0, 7.1)	3.5 (0.0, 8.8)	6.4 (0.0, 12.8)	0 (––)

42 % of patients were diagnosed with Depressive disorders. Among these patients, self-harm behavior was reported by 40.5 % of patients (n = 17) and suicidality was reported by 64.3 % of patients (n = 27). 23 % of patients were diagnosed with Bipolar and related disorders. None of these patients reported self-harm behavior; suicidality was reported by 13 % of these patients (n = 3). 21 % of patients were diagnosed with Anxiety disorders, Obsessive Compulsive disorder, Trauma and stress related disorders. Among these patients, self-harm behavior was reported by 14.3 % of patients (n = 3) and suicidality was reported by 19 % of the sample (n = 4). Only 13 % of patients (n = 13) were diagnosed with Schizophrenia spectrum and other psychotic disorders; due to the small sample size these patients were excluded from the analyses performed on each diagnostic group. Although a diagnosis of psychotic disorders was infrequent in our sample, about 29 % of our sample, across the different diagnostic groups, presented with psychotic features in their symptomatology. Self-harm behavior was reported by 10.3 % of these patients; suicidality was reported by 20.7 % of these patients.

Looking at the relationship between clinical diagnosis and residential status, no significant difference was observed between Emirati and non-Emirati patients in the frequency of Depressive disorders (*χ*^2^ = 0.15; p = 0.70), Bipolar and related disorders (*χ*^2^ = 0.84; p = 0.36), or Anxiety and related disorders (*χ*^2^ = 2.23; p = 0.14). Depressive disorders were more common among female help-seekers than males (*χ*^2^ = 3.93; p < 0.05). The frequency of Anxiety disorders and Bipolar and related disorders did not significantly differ between males and females (Anxiety: *χ*^2^ = 1.08; p = 0.30; Bipolar: *χ*^2^ = 1.17; p = 0.28).

The results of the negative binomial regression, conducted on the duration of symptoms before seeking help, indicated that Family Stress was a significant predictor of symptoms duration (IRR = 1.75; 95 % CI = 1.02-3.00; p < 0.05). Specifically, the number of days before seeking help was 1.75 times greater among those who reported Family Stress compared to individuals who did not report stress related to the relationship with their family. Similarly, the duration of symptoms was significantly associated with Gender (IRR = 0.49; 95 % CI = 0.28–0.88; p < 0.05), indicating that the number of days before seeking help was 0.49 times greater among males compared to females. Self-harm behavior was significantly associated with symptoms duration (IRR = 2.11; 95 % CI = 1.10–4.06; p < 0.05); the number of days before seeking help was 2.11 times greater among individuals who reported self-harm behavior compared to those who didn’t. The presence/absence of psychotic symptoms also significantly predicted symptoms duration (IRR = 0.34; 95 % CI = 0.17–0.67; p < 0.01); the number of days before seeking help was 0.34 times greater among individuals who did not experience psychotic symptoms compared to those who did. Finally, a diagnosis of Anxiety or stress related disorders was significantly associated with duration of symptoms (IRR = 3.19; 95 % CI = 1.10–9.25; p < 0.05); the number of days before seeking help was 3.19 times greater among individuals who received a diagnosis of Anxiety or related disorders compared to those who didn’t. The remaining predictors entered in the model did not have a significant effect on the duration of symptoms before seeking help. These results are presented in Table 4.

**Table 4:** Results of the negative binomial regression

Predictor*	B	S.E.	Wald Chi–Square	Sig.	IRR	95 % CI
Family stress	**0.56**	**0.27**	**4.10**	**< 0.05**	**1.75**	**1.02–3.00**
Friends stress	− 0.40	0.35	1.30	0.25	0.67	0.34–1.33
Academic stress	− 0.21	0.28	0.54	0.46	0.81	0.47–1.41
Gender (Male vs. Female)	**− 0.71**	**0.30**	**5.67**	**< 0.05**	**0.49**	**0.28–0.88**
Residential status	0.13	0.28	0.21	0.64	1.14	0.65–1.98
Self–harm	**0.75**	**0.33**	**5.06**	**< 0.05**	**2.11**	**1.10–4.06**
Suicidality	0.33	0.35	0.93	0.33	1.40	0.71–2.75
Psychotic features	**− 1.09**	**0.35**	**9.48**	**< 0.10**	**0.34**	**0.17–0.67**
Depression	0.44	0.58	0.58	0.44	1.56	0.50–4.88
Bipolar	− 0.11	0.51	0.05	0.83	0.89	0.33–2.45
Anxiety	**1.16**	**0.54**	**4.58**	**< 0.05**	**3.19**	**1.10–9.25**

*All predictors compare Yes = 1 versus No = 0 with the exception of Gender where Male = 1 and Female = 2

## Discussion

There is general agreement on the importance of identifying youth mental health needs to improve preventive strategies and early intervention [2]. Despite the progress achieved over the past two decades, the number of studies conducted in the Arab world is scarce. The present investigation aimed to describe a sample of youth accessing hospital-based mental health services in Dubai.

This study highlighted a number of key findings. First, we observed a high prevalence of depressive disorders in our help-seeking sample; this is in agreement with previous studies conducted in the UAE [22, 23]. Furthermore, patients diagnosed with depressive disorders, compared to other diagnostic groups, seemed to have the highest reported frequency of self-harm and suicidality. Self-harm and suicidality are common among young people with mental ill-health, but especially among individuals with mood disorders [33–35]. Importantly, in the present study the small sample size did not allow us to perform statistical comparisons between diagnostic groups, therefore no firm conclusion can be drawn about the relationship between self-harm, suicidality and depressive disorders.

In the overall sample, family stress was prevalent, confirming the well-known link between poor family relationships and mental health trajectories for children and adolescents [36–39]. Non-Emirati patients were more likely than Emirati patients to report their relationships with friends as a source of stress. Notably, our non-Emirati sample included individuals who were born in Dubai as well as those who had previously lived in one or more countries. While friendship patterns have not been directly studied in the UAE, perhaps some considerations can be made, based on descriptions of social and cultural norms at large. On one hand, it is possible to infer that UAE conservative societal norms might restrict the development of intimate friendships outside the family and this could limit the significance of social relationships, along with any stress in relation to it. Although this is a mere speculation, it would be in agreement with studies showing how patterns in friendship development and maintenance tend to reflect societal norms and blueprints [40]. On the other hand, our results could reflect the significance of friendships for expatriate and migrant adjustment and social stress, in agreement with evidence highlighting the importance of peer support for youth living in a foreign country [17, 18].

Family stress was reported more frequently by females than males; this result is consistent with a previous study that explored family functioning and help-seeking behavior, and reported that adolescent females, compared to males, are more likely to report high-conflict within their family [41]. Furthermore, looking at the ethnic compositions of our sample, it seems possible to speculate that this result was at least in part due to cultural norms relating to gender roles and expectations [42]. Nevertheless, our sample size did not allow a direct comparison between different ethnic groups regarding perceived family stressors, and therefore this hypothesis cannot be definitively stated.

Among our treatment-seeking youth, depressive disorders were more common among female patients than males. This is well-established in the literature (e.g. [23, 43]) and possibly reflects gender differences in help seeking behavior [44]. Indeed, the reluctance of men to seek help has been linked to societal norms regarding the traditional masculine gender role, possibly exerting pressure for conformity and inhibiting willingness and ability to seek help [45]. Notably, our sample was young, and in most cases the decision to seek professional help was taken by parents or relatives. According to Eapen and Ghubash [27], in the UAE, girls with emotional problems have a better chance of getting professional help compared to boys, due to parents not recognizing boys’ problems as needing medical help; this would lead to an overall lower utilization of psychiatric services for boys compared to girls [46].

Consistent with this finding, the results of the regression analysis in this study indicated longer duration of symptoms before help-seeking among male patients compared to females. Family stress was also associated with longer symptoms duration. As stated above, with family stress being prevalent in our sample, and parents often being key initiators of the help-seeking process, this result is not unexpected. Notably, stressful experiences related to the family context often included absence of a parent or difficult relationship with one or more member of the family (see Table 1); having an absent, deceased, abusive, or mentally ill parent could have contributed to delaying help-seeking in our sample. This hypothesis, however, needs to be further address by future research.

The presence of psychotic features in patients’ symptomatology was associated with faster help seeking. In other words, patients presenting with psychotic features were, generally, seeking professional help sooner. This could reflect the perceived seriousness of the symptoms. Accordingly, Biddle and colleagues [47] looked at help seeking choices among young adults and found that individuals distinguished between ‘normal’ and ‘real’ distress, where ‘real’ distress, including psychosis, schizophrenia, and other unusual types of depression, was described as outward, visible, disabling, and pervasive. This is also in keeping with evidence demonstrating that the more outward and visible the symptoms, the faster patients seek specialized care [48]. On the other hand, these results also highlight the need for interventions promoting faster access to medical care for individuals experiencing non-psychotic symptoms. In addition to setting up early intervention services, awareness programs geared towards educating young people and their families on identifying the early signs of illness may assist in early detection.

On the other hand, self-harm significantly predicted longer duration of symptoms, in line with previous studies (e.g., [49]). It is possible to suggest that patients might have used self-harm as a strategy to temporarily cope with mental illness and delay help seeking. This would be consistent with what has been reported in previous studies [50]. It has also been reported that stigma against self-harm creates a barrier to help seeking [51]. Both the affect regulation and stigma hypotheses might have contributed to delaying help-seeking behavior in our sample.

Lastly, longer duration of symptoms was associated with a diagnosis of anxiety disorders, a pattern that is also well-established in the literature (e.g., [52]). Previous studies have highlighted several barriers to accessing medical treatment for individuals affected by anxiety disorders, including the cost of treatment, the belief that individuals should be able to manage their anxiety problems on their own, and the tendency to seek help among friends or at home rather than in professional settings, among others [53]. Due to lack of statistical power, our data does not allow further exploration of the relationship between symptoms duration and anxiety disorders. Larger and longitudinal studies could help clarify the nature of the relationship between duration of symptoms and anxiety disorders as well as the contribution of cultural factors and immigration status on help-seeking choices for youth with anxiety disorders.

### Limitations

To the best of our knowledge, this was the first study attempting to characterize youth who access mental health services in Dubai. Our study included different ethnic groups, reflecting Dubai’s multi-cultural environment. On the other hand, the ethnic composition of our sample is not representative of the population of Dubai residents. Furthermore, our sample was relatively small and selective, although it must be noted that the data was collected from the archives of Rashid Hospital. As previously mentioned, Rashid Hospital has the main Emergency Department serving Dubai and the northern Emirates. It is also worth noting that it is a common practice in the UAE to seek medical treatment (including mental health services) abroad or from private institutions to avoid stigma. Therefore, it is possible that our sample largely represented a specific socioeconomic subgroup of the population who did not have the means to seek treatment abroad or from private clinics. Another limitation of the present work is that it is mainly based on self-reported information (collected from patients or family members), therefore it is possible that our data lacks objectivity or that some relevant information (e.g., substance abuse) might have been omitted due to its legal implications. Notably, due to the nature and composition of our population, performing standardized mental health assessments is often challenging. This is in part due to the shortage of standardized measures translated and validated in Arabic language. Finally, an important limitation of the present study is that it did not capture those who never seek medical treatment. Furthermore, due to our specific focus on youth, our results cannot be generalized to those who start experiencing mental ill-health in youth but only seek medical treatment later in adulthood. Indeed, it is a common practice in the UAE, and in Arab countries in general, for individuals to first seek support for mental health issues from religious and/or traditional leaders, and to only seek referral to mental health services as a last resort.

### Future directions

In line with health initiatives promoted worldwide, the Dubai government has identified mental health as one of the areas requiring attention and has approved a strategic plan supporting mental health research, education and promotion with a specific focus on youth (Dubai Mental Health Strategy 2021). Approved by The Executive Council in 2018, the Dubai Mental Health Strategy’s mission is to ‘*promote mental well-being and provide accessible, high quality, and evidence-based services within an integrated, comprehensive, and culturally appropriate system’*. Since then, remarkable progress was achieved in awareness, recognition and treatment of mental disorders. Nevertheless, some future recommendations can be made. Sewilam and colleagues have highlighted the need, for countries of the Middle East, to reduce stigma, to improve the psychiatrist:population ratio, and to promote the dialogue between mental health professionals and traditional healers [54]. Moreover, some studies conducted in the UAE have highlighted the need to enhance mental health literacy among school nurses and pediatric health staff [55, 56]. The shortage of standardized measures translated and validated in Arabic language also needs to be addressed. Although the majority of individuals living in the UAE are bilingual (often even multilingual), mental health assessment in the patient’s preferred language is warranted (e.g., [57]). Furthermore, adapting assessment methods and responding to the needs of individuals of diverse cultural, social and religious background can be challenging for service providers. Indeed, proficiency in intercultural communication and treatment has been identified as a global target for psychiatry training [58]. A global and integrated mental health approach is crucial to improve life trajectories, especially so in modern multi-cultural societies [10, 59].

## Conclusions

The present study contributes to characterizing youth accessing mental health services at Rashid Hospital’s Child Psychiatry service in Dubai. Our sample of help-seekers included a roughly equal number of Emiratis and expatriates from both genders, although the number of females was slightly greater than males. An overall prevalence of poor family functioning was observed, and the importance of peer support for expatriate help-seekers was highlighted. Gender differences in perceived stressors, clinical diagnoses and help-seeking behavior, possibly due to cultural norms, suggest the need to further promote help-seeking among young boys. For about one third of our help-seeking sample, patients’ presenting symptomatology included psychotic features; these individuals reported a faster access to mental health services; on the other hand, help-seeking was delayed in those reporting self-harm behavior and in those with a diagnosis of Anxiety or related disorders. Of note, self-harm and suicidality were frequently reported in our sample, possibly highlighting the contingency of help-seeking on the perceived severity of the symptoms. This hypothesis, however, needs to be further investigated. Overall, these findings help better understand mental health needs and pathways to care for youth living in Dubai. The findings provide a baseline for future community-based studies, and may also lay the groundwork for larger multi-centre studies across the Emirates. This would potentially contribute to the planning of targeted preventive strategies to improve life trajectories for youth living in the UAE, in line with governmental strategic plans.

## Data Availability

The datasets used and/or analyzed during the current study are available from the corresponding author on reasonable request.
